# Spontaneous brain activity following fear reminder of fear conditioning by using resting-state functional MRI

**DOI:** 10.1038/srep16701

**Published:** 2015-11-18

**Authors:** Pan Feng, Yong Zheng, Tingyong Feng

**Affiliations:** 1 Faculty of Psychology, Southwest University, Chongqing 400715, China; 2 Key Laboratory of Cognition and Personality (SWU), Ministry of Education, Chongqing 400715, China

## Abstract

Although disrupting reconsolidation may be a promising approach to attenuate or erase the expression of fear memory, it is not clear how the neural state following fear reminder contribute to the following fear extinction. To address this question, we used resting-state functional magnetic resonance imaging (rs-fMRI) to measure spontaneous neuronal activity and functional connectivity (RSFC) following fear reminder. Some brain regions such as dorsal anterior cingulate (dACC) and ventromedial prefrontal cortex (vmPFC) showed increased amplitude of LFF (ALFF) in the fear reminder group than the no reminder group following fear reminder. More importantly, there was much stronger functional connectivity between the amygdala and vmPFC in the fear reminder group than those in the no reminder group. These findings suggest that the strong functional connectivity between vmPFC and amygdala following a fear reminder could serve as a key role in the followed-up fear extinction stages, which may contribute to the erasing of fear memory.

Fear conditioning plays an important role in the development of a wide range of psychopathologies including anxiety, phobia, and posttraumatic stress disorder (PTSD)^1^,^2^. In an attempt to identify the neural network of fear processes, numerous neuroimaging studies have elucidated the neural systems of fear processes using the paradigm of classical fear conditioning^3^,^4^,^5^,^6^,^7^,^8^,^9^. The fear processes can be divided into four distinct phases: fear acquisition, fear consolidation, fear reconsolidation, and fear extinction^2^,^10^,^11^. However, little is known about functional connectivity and spontaneous neuronal activity following a fear reminder. It is particularly unclear how the fear reminder contributes to the next fear extinction stage. Studies of Pavlovian fear conditioning in rodents and humans indicate that a neural circuit including the amygdala, insula, dorsal anterior cingulate (dACC), prefrontal cortex, and temporal cortex was involved in fear acquisition^12^,^13^,^14^. With regard to fear consolidation, considerable evidence indicate that amygdala, parahippocampus and amygdala-dmPFC, amygdala-dACC, and amygdala-mPFC functional connectivity were critical for the fear memory consolidation^4^,^9^. As for fear extinction, cumulative evidence have identified the amygdala, hippocampus (Hip), ventromedial prefrontal cortex (vmPFC), and dorsal lateral prefrontal cortex (dlPFC) as the major regions involved in fear extinction^15^,^16^,^17^,^18^,^19^,^20^. Specifically, Phelps *et al*. found that amygdala activation was correlated across subjects with the conditioned response in both acquisition and early extinction, suggesting that amygdala response may be related to acquisition learning, extinction learning and early extinction success. Moreover, vmPFC (subgenual anterior cingulate) activation at the beginning stage of day 2 extinction was significantly correlated with day1 extinction success, indicating that the vmPFC is critical for the retention of extinction^6^. Furthermore, Delgado *et al*. found that dlPFC activation was correlated across subjects with emotion regulation success and the left amygdala and dlPFC were positively correlated with the vmPFC in functional connectivity analysis. These results suggest that the lateral PFC regions engaged in cognitive emotion regulation strategies may influence the amygdala, diminishing fear through similar vmPFC connections that are thought to inhibit the amygdala during extinction^20^.

Many recent behavioral studies of fear reconsolidation demonstrate that fear extinction conducted following a fear reminder can prevent the spontaneous recovery^2^,^10^,^21^,^22^. Moreover, recent research in rodents suggested that the amygdala and the vmPFC played an important role in the reconsolidation of emotional memory traces following their retrieval^23^,^24^. However, why does the fear reminder contribute to the following fear extinction? Past researches provided a couple of explanations: the enhancement of extinction and the disruption of reconsolidation. In terms of enhancement of extinction, the fear reminder was thought to enhance the formation of a new conditioned stimulus (CS)-no-event association that competes with the original CS-US memory trace^25^,^26^,^27^. However, disruption of reconsolidation was supposed to weaken or erase the original association between the CS and the US (the subject forgets that the CS ever predicted the US)^28^,^29^,^30^. The present study was conducted to investigate whether the extinction effect was due to the enhancement of extinction or disruption of reconsolidation.

RSFC is a powerful approach for understanding composition and stability of these functional networks^31^,^32^,^33^,^34^. Regarding the amplitude of LFF(ALFF), Biswal *et al*. found that ALFF was higher in grey matter than in white matter^35^. In addition, Kiviniemi *et al*. reported activation in the visual cortex due to low-frequency fluctuations at about 0.034 Hz using the power spectrum method^36^. Moreover, Zang *et al*. found that ADHD children have different ALFF in some brain areas, indicating that ALFF may be a potential index of regional spontaneous neuronal activity^37^. By using RSFC and ALFF, the present study attempted to examine the resting state of brain (functional connectivity and spontaneous neuronal activity) following the fear reminder.

Based on the studies mentioned above^2^,^10^,^23^,^24^, we hypothesized that there would be equal fear ratings between the reminder group and the no reminder group at the fear acquisition (on the first day) and extinction stages (on the second day). However, at the re-extinction stages (on the third day), fear ratings of the no reminder group would be greater than these of the reminder group. Moreover, we also expected to observe different spontaneous neuronal activities and functional connectivities between the reminder group and the no reminder group following fear reminder (on the second day). Additionally, we expected to observe activation of the fear matrix that includes amygdala, dACC, insula, thalamus, and temporal lobe, in the stage of acquisition. Moreover, we also expected to observe different brain activities between the reminder group and no reminder group in the stage of re-extinction. Specifically, dlPFC activation is hypothesized to emerge in the reminder group, while the activity of the vmPFC and amygdala would be greater in the no reminder group.

## Results

### Behavioral result

Subjective fear ratings (CS+ and CS−) were acquired using an 1–7scale of fearfulness (on a 7-point Likert scale: 1, a little; 4, moderately; 7, extremely) at the stage of fear acquisition (on the first day), fear memory extinction (on the second day) and fear memory re-extinction (on the third day). On the first day, two-way mixed ANOVA analysis on the group (fear acquisition group vs. control group) and the type of the conditioned stimulus (CS+ vs. CS−) revealed that there was a significant interaction between two factors, F(1,54) = 79.45, p < 0.001. To examine the effect of experiment treatment, we performed simple effect analysis. In the fear acquisition group, the subjective fear ratings of CS+ and CS− was as follows: the CS+: *M* = 5.42, *SD* = 1.52, the CS−: *M* = 1.28, *SD* = 0.77, *t* (35) = 13.07, p < 0.001. In the control group, the subjective fear ratings of CS+ and CS− were as follows: the CS+: *M* = 1.75, *SD* = 0.55, the CS−: *M* = 1.6, *SD* = 0.60, *t* (19) = 1.14, p = 0.27. For another simple effect analysis, the following results were obtained: there was no significant difference between the subjective fear ratings of CS− in fear acquisition group (M = 1.28, SD = 0.77) and that in the control group (M = 1.6, SD = 0.60), t(54) = 1.59, p = 0.12; However, there was greater subjective fear ratings of CS+ in the fear acquisition group (M= 5.42, SD = 1.52) than that in the control group (M = 1.75, SD = 0.55), t(54) = 10.34, p < 0.001.

In the following analysis, we used the differential fear ratings (CS+ substract CS−) as the fear indicator which was in line with the prior researches^2^,^10^,^38^. Additionally, there was no significant difference in the subjective fear ratings (CS+ substract CS−) between the reminder group (M= 4.45, SD = 1.19) and the no reminder group (M = 4.13, SD = 1.09) during the stage of fear acquisition (on the first day), t(34) = 0.845, p = 0.40. The behavioral results indicated that the fear acquisition group (the reminder group and the no reminder group for second-day study) acquired the conditioned fear.

**Figure 1:**
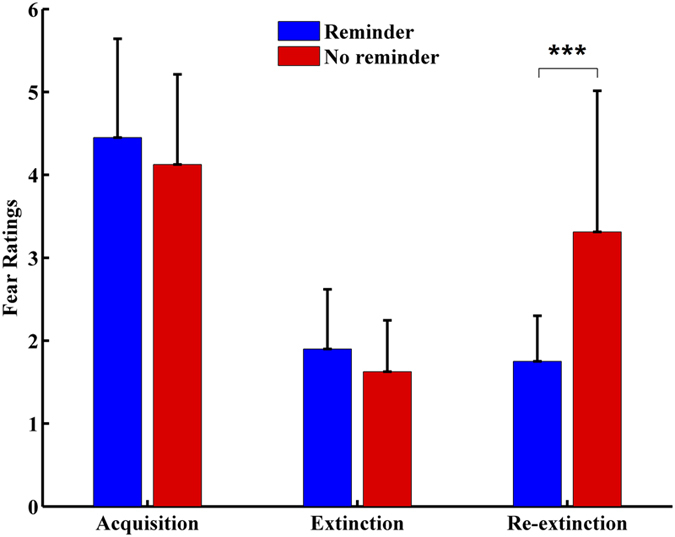
Mean differential fear ratings (CS+ vs. CS−) during acquisition, extinction and re-extinction stages in the reminder group and the no reminder group.

Extinction effect was evaluated using a two-way mixed ANOVA on the group (reminder group vs. the no reminder group) and the time (the second day vs. the third day). The results showed a significant interaction between the group and time, F(1, 34) = 14.18, *p* < 0.001. Thus, we performed an simple effect analysis, and the results indicated that there was no significant difference in fear ratings (the differential fear ratings between CS+ and CS−) between the reminder group (M = 1.90, SD = 0.72) and the no-reminder group (M = 1.63, SD = 0.62) during the stage of fear extinction (on the second day) (p = 0.23). Subjects in the no-reminder group had greater fear ratings (M = 3.31, SD = 1.70) than those in the reminder group (M = 1.75, SD = 0.55)(on the third day) (p = 0.001). For another simple effect analysis, the following results were observed: there was no significant difference in subjective fear ratings of the reminder group between the second day (M = 1.90, SD = 0.72) and the third day (M = 1.75, SD = 0.55) (p = 0.42); However, in the no reminder group, subjects had greater fear ratings M = 3.31, SD = 1.70) on the third day than that on the second day (M = 1.63, SD = 0.62) (p < 0.005) (see Fig. 1).

In summary, the findings are consistent with those from prior research^2^,^10^. The behavioral results indicated that spontaneous recovery appeared only in the no reminder group on the re-extinction stages, but there was no spontaneous recovery in the reminder group. In addition, our behavioral results demonstrated that extinction during fear reconsolidation can prevent the spontaneous recovery of fear following the fear memory reminder.

## Resting-state fMRI Results

### The ALFF result following the fear reminder

**Figure 2:**
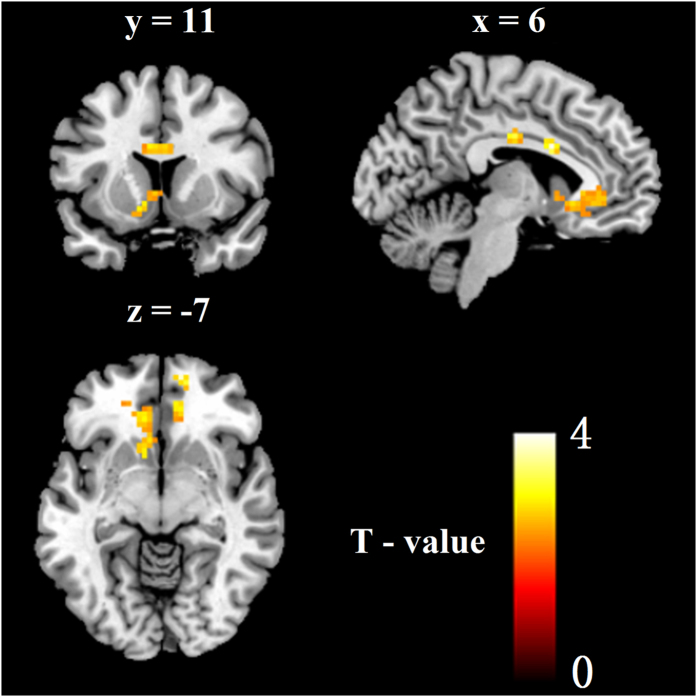
ALFF differences between reminder group and the no reminder group following the fear reminder. (P < 0.01, *Alphasim corrected*; Voxels  ≥ 40).

In order to investigate spontaneous brain activity following fear memory reactivation, a two sample t-test (ALFF image for each group) was conducted. The results indicated significant difference between the two groups in some brain areas. Areas showing increased ALFF in the reminder group included dACC and vmPFC(t[36] = 2.43, p = 0.01)(see Fig. 2 Table 1). The difference of brain activity between two groups indicated dACC may play a crucial role in fear expression^7^,^39^. More importantly, the results also suggest that the vmPFC may serve a key role in the modulation of the conditioned fear following fear reminder^23^.

### The RSFC result (voxel wise) following fear reminder

**Figure 3:**
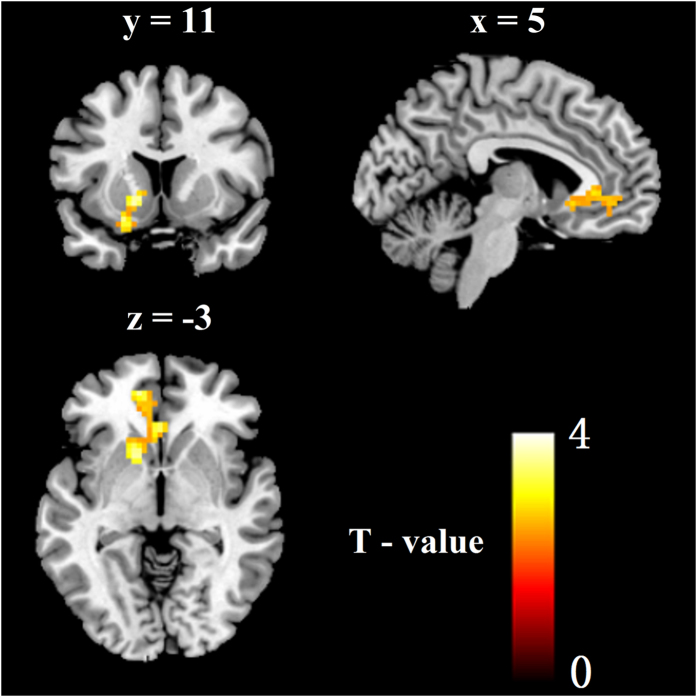
There was much stronger functional connectivity between the amygdala and vmPFC in the reminder group than that in the no reminder group following the fear reminder. (P < 0.01, *Alphasim corrected*; Voxels ≥ 40).

To further investigate functional networks after the fear reminder, we performed a two sample t-test analysis (voxel wise image for each group) on rs-fMRI data. The results indicated that there was much stronger functional connectivity between the amygdala and vmPFC in the reminder group than that in the no reminder group (t[36] = 2.43, p = 0.01) (see Fig. 3). Furthermore, the results suggested that the RSFC between amygdala and vmPFC may play an important role in the state of fear memory reactivation, which may influence the following extinction effect.

In the stage of fear acquisition, we found that the fear matrix, including amygdala, dACC, mPFC, insula, thalamus and temporal lobe, is only active in the experimental group (the reminder group and no reminder group), but not in control group. Moreover, on day1 the experiment began with a baseline rest condition (REST1, 10 min), then participants completed fear acquisition task (40 min) before the experimental rest condition (REST2, 10 min)^9^,^11^. To verify whether the ALFF and functional connectivity difference between two groups were due to fear reminder, we performed two sample t-test of REST1(REST1 for reminder group vs. REST1 for no reminder group) and REST2(REST2 for reminder group vs. REST2 for no reminder group) in two groups respectively. The result revealed that there were no differences of ALFF and functional connectivity between two groups on day1 before and after fear conditioning. The results suggest that the observed ALFF and functional connectivity differences between two groups due to fear reminder.

In summary, the ALFF and the RSFC results suggest that the amygdala and the vmPFC served a key role in the state of fear memory reactivation following fear memory retrieval. However, could spontaneous neuronal activity and the RSFC contribute to the following extinction stages? These findings suggest spontaneous neuronal activity and the RSFC following fear reminder may play an important role in the following extinction stages, which may contribute to erase the fear memory.

### Brain–behavior correlation results

**Figure 4:**
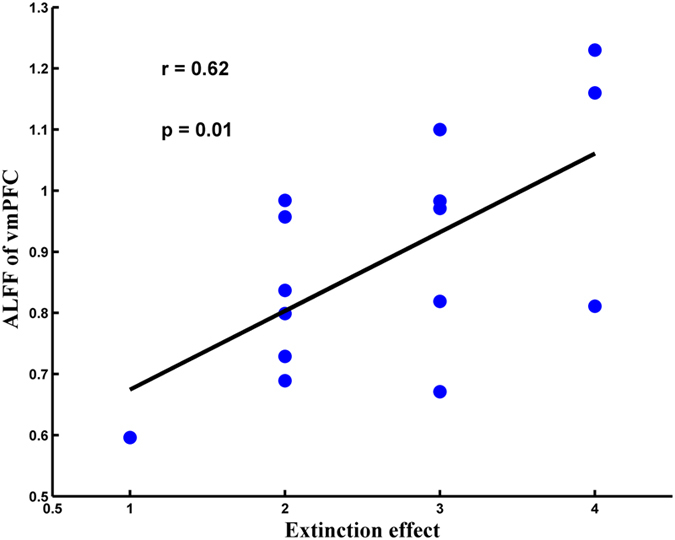
The ALFF of vmPFC was positively correlated with the change (∆) in subjective fear ratings in the reminder group (*r* = 0.62, *p* = 0.01).

To examine whether the ALFF following fear reminder predicts extinction effect, we conducted the correlation analysis between ALFF and the change (∆) in subjective fear ratings in the reminder group. The results showed that the ALFF of vmPFC was positively correlated with the change (∆) in subjective fear ratings (*r* = 0.62, *p* = 0.01) (see Fig. 4). The findings suggest individual differences in ALFF following fear reminder can predict individuals’ extinction effect.

## Discussion

Using ALFF and RSFC, we investigated the spontaneous neuronal activities and functional networks following fear memory reminder. The behavioral results showed that there were no significant differences of fear ratings between the reminder group and the no reminder group at the fear acquisition and extinction stages, but spontaneous recovery appeared in the no reminder group at re-extinction stages only. Moreover, neuroimaging results yield two dominating findings. Firstly, after the fear reminder, we found some brain regions including dACC and vmPFC showed greater activations in the fear reminder group than that in the no reminder group. Secondly, there was much stronger functional connectivity between the amygdala and vmPFC in the reminder group than that in the no reminder group. In summary, these results suggest that, following fear reminder, stronger functional connectivity between vmPFC and amygdala in reminder group could serve a key role in the following fear extinction stages, which may contribute to the erasing of the fear memory.

In consistent with prior researches, our behavioral results showed that there was no significant differences in fear ratings between the reminder group and the no reminder group at the fear acquisition and extinction stages, but spontaneous recovery appeared in the no reminder group at re-extinction stages (disruption of reconsolidation). In the present study, we performed the extinction during the fear memory reconsolidation window, which may disrupt fear memory reconsolidation (attenuated, or erasure the original CS-US memory trace). Disruption of reconsolidation was regarded to weaken the original association between the CS and the US (i.e., the subject forgets that the CS ever predicted the US^28^,^29^,^30^). Moreover, it was also suggested that the reminder and the fear reconsolidation window may be important determinants to prevent spontaneous recovery^10^,^40^,^41^.

With respect to the neural systems following the fear reminder, the first finding was that some brain areas including vmPFC and dACC showed increased ALFF in the reminder group than that in the no reminder group. Several human neuroimaging studies have shown that the dACC were closely associated with fear acquisition and the expression^7^,^42^,^43^,^44^. In the present study, our findings indicate that the dACC served a key role in the expression of conditioned fear responses following the fear memory reminder. In addition, regulatory mechanisms such as reappraisal, suppression, and regulation, were thought to reside in the PFC. Specifically, a wealth of studies have suggested that ventral-rostral portions of the ACC and mPFC play a regulatory role in limbic regions involved in generating emotional responses^39^,^45^,^46^,^47^. Moreover, the vmPFC may play an important role in the ‘‘automatic’’ emotion regulation processes^48^,^49^,^50^. In our study, the vmPFC might function through dampening the output of other brain regions involved in conditioned fear response, such as amygdala, dACC.

Importantly, we mapped the amygdala circuitry in humans using RSFC techniques, which rely on detecting coherent patterns of spontaneous activities and delineate entire functional networks^51^. The result showed that there was were much stronger functional connectivity between the amygdala and vmPFC in the reminder group than that in the no reminder group. Prior studies have demonstrated that the amygdala and vmPFC served a key role in the fear reconsolidation process. Specifically, Tronson and his fellows suggested that activation of amygdala PKA enhanced fear reconsolidation process only when the fear memory was retrieved and PKA inhibition impaired reconsolidation^52^. Moreover, the protein synthesis of the amygdala was also indispensable for fear memory reconsolidation process following the fear reminder^24^. Regarding the vmPFC, protein synthesis and NMDA receptors played an important role in fear reconsolidation^23^. Regarding functional connectivity between amygdala and vmPFC, Agren *et al*. found that there was stronger functional connectivity between amygdala and the fear network (insula, hippocampus, and the midline anterior cingulate cortex) after normal memory reconsolidation but not after disrupted reconsolidation^38^. Moreover, Schiller *et al*. found that extinction during fear memory reconsolidation window diminished the involvement of the vmPFC. The results showed that there was stronger functional connectivity between the vmPFC and amygdala during extinction of the non reminded, but not the reminded CS+^53^. A lot of studies led to the establishment of the reconsolidation theory, which assumed that a short CS− alone trial leads to memory reminder that activates a temporary labile state of the memory, during which the memory, i.e., the original CS-US association, can be modified, strengthened or attenuated/erased, before reconsolidating and becoming stable again^28^,^29^,^30^. In the present study, we performed the extinction during the fear memory reconsolidation window, which disrupted fear memory reconsolidation (attenuated, or erasure the original CS-US memory trace). Disruption of reconsolidation is regarded to weaken the original association between the CS and the US, i.e., the subject forgets that the CS ever predicted the US^28^,^29^,^30^. In the present study, disruption of reconsolidation may prevent the protein synthesis and NMDA/PKA activation in the amygdala and vmPFC. Moreover, extinction during the fear memory reconsolidation window may alter functional connectivity between the amygdala and vmPFC. Different from the no reminder group, there may be a relative disconnect between the amygdala and vmPFC at fear extinction stage in the reminder group. This altered connectivity may play an important role in enabling extinction learning to more permanently modify the original fear memory trace^53^. As a result, spontaneous recovery during re-extinction appeared only in the no reminder group, but not in the reminder group (disruption of reconsolidation). Simpson *et al*. found that the Regional cerebral blood flow (BF) of mPFC decreases in these areas were inversely correlated with self-rated anxiety, such that the least anxious subjects exhibited the largest BF reductions, whereas the most anxious subjects showed no significant BF reduction or a slight increase^54^. Regarding the therapy for PTSD patients, recent studies show that the combination of reconsolidation and extinction approaches may be more efficient in eliminating learned fear than using extinction alone^2^,^10^. However, exposure therapy, functions differently through augmenting reconsolidation of trauma-related memories in the context of associated arousal and an increase in noradrenergic signaling^55^. In our study, greater connectivity of the default network (PCC, mPFC, precuneus) such as vmPFC with the amygdala after fear reminder may be particularly interesting. These findings suggest that a function of the default network is to maintain the organism in a state of readiness for expected future events^56^.

Interestingly, the ALFF of vmPFC was positively correlated with the change (∆) of subjective fear ratings. Motzkin *et al*. have found that vmPFC lesions were associated with increased right amygdala reactivity to aversive stimuli and increased resting state connectivity with anterior temporal cortex. It suggests that it is a critical role for the vmPFC in regulating amygdala activity^57^. Among health adults, greater functional connectivity between the right amygdala and bilateral IFG, OFC, vmPFC, anterior cingulate cortex, and frontopolar cortex was associated with threat exposure^58^. These findings are directly relevant to neural circuitry models of emotion regulation and affective psychopathology. In our study, exposure to fear reminder modulates ALFF of vmPFC and amygdala-vmPFC functional connectivity. This may help to maintain extinction effect when individuals are experiencing anxiety induced by fear reminder. That explains the reason why the ALFF of vmPFC following the fear reminder is able to predict the extinction effect.

The present study examined the resting state of brain (functional networks and spontaneous neuronal activity) following the fear reminder using RSFC and ALFF. Specifically, some brain regions including dACC and vmPFC showed greater activations in the fear reminder group than those in the no reminder group. Moreover, there was much stronger functional connectivity between the amygdala and vmPFC in the reminder group than that in the no reminder group. Accordingly, the present study provided insight into functional networks and spontaneous neuronal activities after the fear memory activation. The present study also provides an explanation for why extinction conducted following the fear reminder can prevent the spontaneous recovery process (disruption of reconsolidation). However, the current study used self-rated fear and did not include widely-used objective behavioral data such as SCR. Findings should be interpreted and generalized with caution.

## Methods

### Subjects

Three groups of participants with no history of neurological or psychiatric disorders were recruited, with each group consisting of 20 college students from a Chinese university: the reminder group (*M*_age_ = 21.60, SD = 1.57 years, 10 females), the no reminder group (*M*_age_ = 22.05, SD = 1.80, 12 females), and the control group (*M*_age_ = 22.05, SD = 1.80, 12 females). There was no significant difference of trait anxiety among the reminder group (M = 41.4, SD = 1.85), the no reminder group (M = 40.45, SD = 1.86) and the control group (M = 42.1, SD = 1.82), F(2, 57) = 0.20, p = 0.82. There was also no significant difference with regard to the state anxiety among the reminder group (M = 36.55, SD = 1.59), the no reminder group (M = 34.70, SD = 1.40) and the control group (M = 34.75, SD = 1.40), F(2, 57) = 0.52, p = 0.60. All participants were provided written informed consent and paid for their participation. The study were approved by the local ethics committee of Southwest University and the Institutional Human Participants Review Board of the Southwest University Imaging Center for Brain Research. The methods were carried out in accordance with the approved guidelines. The resting-state stages (reconsolidation stages) were performed in the scanner at the second day, and two participants in the no reminder group were removed from the resting-state analysis due to excessive head movement.

### Stimuli

As the material of US and the neutral stimulus, 533 pictures (the fear pictures and neutral pictures) chosen from the Internet and International Affective Picture System (IAPS) were used in the fear acquisition, extinction and re-extinction task^9^,^59^. Other fifty participants rated 533 pictures. In accordance with the previous research, we used a dimensional model for measuring pictures along 3 dimensions: ‘‘valence’’, ‘‘arousal’’ and ‘‘the degree of fear’’. They rated the respective dimensions on a 7-point Likert scale. Finally we chose 60 fear pictures and 160 neutral pictures, in which the disparity of the degree of fear and the valence was as large as possible (the fear picture (fear): *M* = 5.72, *SD* = 0.51, the neutral picture (fear): *M* = 1.85, *SD* = 0.55, *t*(49) = 33.38, *p* < 0.001; the fear picture (valence): *M* = 6.08, *SD* = 1.09, the neutral picture (valence): *M* = 2.86, *SD* = 0.50, *t*(49) = 18.35, *p* < 0.001 and the arousal of fear pictures and neural pictures was as follows: the fear picture (arousal): *M* = 5.12, *SD* = 0.35, the neutral picture (arousal): *M* = 4.74, *SD* = 0.27, *t*(49) = 1.36, *p* > 0.1. The conditioned stimulus (CS+, CS−) were yellow and blue squares and the unconditioned stimulus (US), and the neutral stimulus were the fear pictures and neutral pictures respectively.

### Design and Procedure

**Figure 5:**
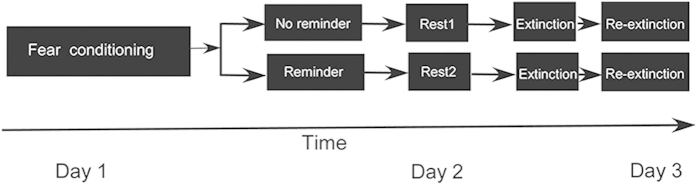
Diagram of the phases in the experiment.

The experiment consisted of three stages that took place on three consecutive days: Day 1 -Acquisition, Day 2-Reminder, Reconsolidation and Extinction, and Day 3 - Re-extinction (see Fig. 5). The detailed procedures are described below:

Day 1: fear acquisition

All fear acquisition participants (n = 40) underwent a Pavlovian discrimination fear-conditioning paradigm with partial reinforcement at the fear acquisition stage^2^,^10^,^38^, while the control group (n = 20) underwent the same task without reinforcement. Two stimuli consisting of squares either in blue or yellow color signaled the presence (CS+) or absence (CS−) of a fear picture. The conditioned stimuli was presented for 2 s, and the inter-trial-interval (ITI) was presented for 2–6 s. The CS+ was paired with the fear picture on a 62.5% partial reinforcement schedule and the CS− was always paired with neutral picture. Subjects were instructed to pay attention to the screen and try to figure out the relationship between the squares and the following picture. Moreover, when the CS+ appeared on screen, the subjects need to press key “1”, otherwise they should press “3”. Two orders were used to counterbalance for key (within subjects design) and designations of colored squares (blue or yellow) as CS+ or CS− (between subjects design). In control group, subjects performed an associative learning task where the yellow or blue color was paired with two types of emotionally neutral pictures, i.e., scenes or objects. That is, the subjects were instructed to predict the kind of picture following each of the color square. The whole session lasted for 40 min. Subjective fear ratings (CS+ and CS–) were obtained immediately following acquisition stages using an 1–7 fearfulness scale (1: mildly; 4: moderately; and 7: extremely).

Day 2: fear memory reminder, reconsolidation and extinction

On day 2, the fear acquisition participants were randomly assigned to two groups: the reminder group (n = 20) and the no reminder group (n = 20). In the reminder group, extinction training was performed after 10 min and was thus inside the reconsolidation window following fear memory reminder. During the fear reminder, the CS+ with the unconditioned stimulus was presented^25^ followed by a 10 minutes resting state (REST2 for the reminder group, REST1 for the no reminder group) in which the fear memory was activated^2^,^10^. However, in the no-reminder group, all procedures were the same as the reminder group except that no reinforcement was given (the CS+ with the neural pictures presented). During the resting state, subjects were instructed to keep their eyes closed, relax their mind, and remain motionless as much as possible. The resting-state scan lasted for 600 s. All participants reported that they had not fallen asleep during the scan.

The extinction lasted for 30 min, where 60 CS+ and 60 CS− were presented without reinforcement. Subjective fear ratings (CS+ and CS−) were obtained immediately following fear extinction stages using an 1–7 scale of fearfulness (on a 7-point Likert scale: 1: mildly; 4: moderately; 7: extremely).

Day 3 fear re-extinction

In re-extinction stage, the stimulus was presented without reinforcement (the neural pictures terminated with the CS+). The re-extinction was same for both groups and consisted of stimuli with non reinforcement (60 CS+, 60 CS−). The re-extinction sessions lasted for 30 min, and subjective fear ratings (CS+ and CS–) were obtained immediately following re-extinction stages using an 1–7 scale of fearfulness (on a 7-point Likert scale: 1:mildly; 4:moderately; 7: extremely).

Furthermore, to verify that both the subjective fear ratings and the skin conductance response (SCR) are valid indicator of fear response, we recruited another twenty-five right-handed participants (two subjects were eliminated from statistical analysis because they did not acquire fear conditioning) to conduct the same task of fear acquisition using the subjective fear ratings and SCR as the index of fear. A significant between the subjective fear ratings (differential fear ratings, CS+ vs. CS−) and SCR was observed (*r* = 0.57, *p* = 0.005).

We used the fear picture as the unconditioned stimulus at the stage of fear acquisition, so the process of fear acquisition and the extinction become slower. That also explained, the reason why the number of conditioned stimulus (CS+ and CS–) was more than that in the typical design during the stage of fear acquisition, fear extinction and fear re-extinction in the present design^9^,^11^. Moreover, we used the presentation of CS+ and US to activate the fear memory. This treatment was line with the Duvarci and Nader’s research^25^. The presentation of CS+ and US served as the stimulus of the fear reminder but not reinforcement. Finally, in our study, we also used the CS+ and neutral picture in the stage of reactivation in no reminder group to avoid treatment inconsistencies (neutral in no reminder group vs. fear in the reminder group).

### fMRI acquisition

We used a Siemens TRIO 3.0T MRI scanner (Siemens Magnetom Trio TIM, Erlangen, Germany) to collect functional imaging data. Head movement was restricted using foam cushions. T1-weighted images were recorded with a total of 176 slices at a thickness of 1 mm and in-plane resolution of 0.98 × 0.98 mm(TR = 1900 ms; TE = 2.52 ms; flip angle = 9°; FOV = 250 × 250 mm^2^). An Echo-Planar imaging (EPI) sequence was used for data collection (TR = 2000 ms; TE = 30 ms; flip angle = 90°; FoV = 192 × 192 mm^2^; matrix size = 64 × 64; voxel size = 3 × 3 × 3 mm^3^; interslice skip = 0.99 mm; Slices = 32).

### fMRI data analysis

fMRI data was performed using SPM8^60^. For T2*-weighted images, slice timing was used to correct slice order, the data was realigned to estimate and modify the six parameters of head movement, and first five images were discarded to achieve magnet-steady images. The T1-weighted images were co-registered to the EPI mean images and segmented into white matter, grey matter, and Cerebrospinal fluid (CSF). These images were then normalized to MNI space in 3 × 3 × 3 mm^3^ voxel sizes. The normalized data were spatially smoothed with a Gaussian kernel; the full width at half maximum (FWHM) was specified as 6 × 6 × 6 m^3^. The REST and DPARSF software were further used in rest-state analysis^61^,^62^. After preprocessing, the time series for each voxel was filtered (bandpass, 0.01–0.08 Hz) to remove the effects of very-low-frequency drift and high frequency noise such asrespiratory and heart rhythms^35^,^63^. Next, the filtered time series were transformed to a frequency domain with a fast Fourier transform (FFT) and the power spectrum was then obtained. Finally, the square root was calculated at each frequency of the power spectrum and the averaged square root was obtained across 0.01–0.08 Hz at each voxel. This averaged square root was taken as the ALFF^37^.

### ALFF analysis

After achieving the ALFF images of the reminder group and the no reminder group, we performed two sample t-test analysis between the reminder group and the no reminder group (REST2 vs. REST1) to investigate the spontaneous brain activity following a short period of fear memory reactivation, the threshold of *p* < 0.01, Alphasim corrected, voxels  ≥ 40.

### Regions of interest analysis (voxel wise)

For voxel wise analysis, we calculated functional connectivity with the region of interests (ROI) in amygdala based on the voxel wise. ROI were selected on the basis of prior studies^38^ and the ROI was defined using MarsBaR^64^, in other words, the amygdala was defined as a 10 mm spherical ROI centered on the Montreal Neurological Imaging (MNI) coordinate(right amygdala, 27, 5, −17; left amygdala, −15, −1, −14). The functional connectivity was estimated based on the detrended, filtered, and covariables removed images. The covariables included the six head motion parameters, global mean signal, white matter signal and CSF signal^65^. The global signal is thought to reflect a combination of physiological processes (i.e., cardiac and respiratory fluctuations), and therefore, was treated as covariates to account for such effects^51^,^66^,^67^. The time course of each participant was obtained separately from activation maps, and was then used as regressors in a voxel-based whole-brain correlation analysis. Importantly, the time course from the same voxel was used as a regressor for each participant. After obtaining the functional connectivity maps of the reminder group and the no reminder group, we conducted two sample t-test analysis between the reminder group and the no reminder group to investigate the functional connectivity of brain influenced by the fear reminder, the threshold of *p* < 0.01, Alphasim corrected, voxels ≥ 40.

### Correlation analysis between ALFF and behavioral data

To investigate whether the ALFF following fear reminder predicts extinction effect (∆fear ratings: the fear ratings on the first day subtract the fear ratings on the third day), we conducted the correlation between ALFF and the change (∆) in subjective fear ratings (the first day vs. the third day) in the reminder group. ROIs were selected on the basis of the differences of ALFF between the reminder group and no reminder group in rest functional MRI, that is, centering the ROI on the peak of activation of vmPFC (MNI coordinate) (vmPFC, 9, 54, −12).

## Additional Information

**How to cite this article**: Feng, P. *et al*. Spontaneous brain activity following fear reminder of fear conditioning by using resting-state functional MRI. *Sci. Rep*. **5**, 16701; doi: 10.1038/srep16701 (2015).

## Figures and Tables

**Table 1:** Detailed information for clusters showing group ALFF differences (MNI coordinates).

Anatomical label	BA	Cluster size	Hemisphere	Peak t-value	Peak location
aMCC		109	L	4.26	−9 9 27
pMCC	23		R	3.91	6 −15 33
pMCC	24		L	3.47	−6 −12 33
vmPFC	10	78	R	4.01	12 57 −6
vmPFC			R	3.32	9 42 −9
rACC	32		R	2.65	6 42 6
OFC	11	131	L	3.58	−24 36 −12
Caudate			L	3.21	−12 15 −9
pgACC	32		L	3.19	−9 33 −6
Culmen		52	L	3.28	−30 −60 −33

Abbreviations: BA, Brodmann area; L, Left; R, Right; aMCC, Anterior Middle Cingulate Cortex; pMCC, posterior Middle Cingulate Cortex; vmPFC, ventromedial Prefrontal Cortex; rACC, rostral Anterior Cingulate Cortex; OFC, Orbitofrontal Cortex; pgACC, Pregenual Anterior Cingulate Cortex.

ALFF differences between reminder group and the no reminder group following the fear reminder. (REST2 vs. REST1).

## References

[b1] Shin L. M. & Liberzon I. The neurocircuitry of fear, stress, and anxiety disorders. Neuropsychopharmacology 35, 169–191 (2010). 19625997 10.1038/npp.2009.83 PMC3055419

[b2] Schiller D. . Preventing the return of fear in humans using reconsolidation update mechanisms. Nature 463, 49–53 (2009). 20010606 10.1038/nature08637 PMC3640262

[b3] LeDoux J. E. Synaptic self: How our brains become who we are. (Penguin Group: USA, , 2003).

[b4] Schultz D. H., Balderston N. L. & Helmstetter F. J. Resting-state connectivity of the amygdala is altered following Pavlovian fear conditioning. Frontiers in Human Neuroscience 6, 242 (2012). 22936906 10.3389/fnhum.2012.00242 PMC3426015

[b5] Indovina I., Robbins T. W., Núñez-Elizalde A. O., Dunn B. D. & Bishop S. J. Fear-conditioning mechanisms associated with trait vulnerability to anxiety in humans. Neuron 69, 563–571 (2011). 21315265 10.1016/j.neuron.2010.12.034 PMC3047792

[b6] Phelps E. A., Delgado M. R., Nearing K. I. & LeDoux J. E. Extinction learning in humans: role of the amygdala and vmPFC. Neuron 43, 897–905 (2004). 15363399 10.1016/j.neuron.2004.08.042

[b7] Milad M. R. . A role for the human dorsal anterior cingulate cortex in fear expression. Biological psychiatry 62, 1191–1194 (2007). 17707349 10.1016/j.biopsych.2007.04.032

[b8] Milad M. R. & Quirk G. J. Fear Extinction as a Model for Translational Neuroscience: Ten Years of Progress. Annual Review of Psychology 63, 129 (2012). 10.1146/annurev.psych.121208.131631 PMC4942586 22129456

[b9] Feng T., Feng P. & Chen Z. Altered resting-state brain activity at functional MRI during automatic memory consolidation of fear conditioning. Brain research 1523, 59–67 (2013). 23726994 10.1016/j.brainres.2013.05.039

[b10] Monfils M. H., Cowansage K. K., Klann E. & LeDoux J. E. Extinction-reconsolidation boundaries: key to persistent attenuation of fear memories. Science 324, 951–955 (2009). 19342552 10.1126/science.1167975 PMC3625935

[b11] Feng P., Feng T., Chen Z. & Lei X. Memory consolidation of fear conditioning: Bi-stable amygdala connectivity with dorsal anterior cingulate and medial prefrontal cortex. Social cognitive and affective neuroscience 9, 1730–1737 (2014). 24194579 10.1093/scan/nst170 PMC4221215

[b12] LaBar K. S. & Cabeza R. Cognitive neuroscience of emotional memory. Nature Reviews Neuroscience 7, 54–64 (2006). 16371950 10.1038/nrn1825

[b13] Linnman C., Rougemont-Bücking A., Beucke J. C., Zeffiro T. A. & Milad M. R. Unconditioned responses and functional fear networks in human classical conditioning. Behavioural brain research 221, 237–245 (2011). 21377494 10.1016/j.bbr.2011.02.045 PMC3092385

[b14] Linnman C., Zeidan M. A., Pitman R. K. & Milad M. R. Resting cerebral metabolism correlates with skin conductance and functional brain activation during fear conditioning. Biological Psychology 92, 26–35 (2013). 10.1016/j.biopsycho.2012.03.002 22425559

[b15] Milad M. R. & Quirk G. J. Neurons in medial prefrontal cortex signal memory for fear extinction. Nature 420, 70–74 (2002). 12422216 10.1038/nature01138

[b16] Morgan M. A. & LeDoux J. E. Differential contribution of dorsal and ventral medial prefrontal cortex to the acquisition and extinction of conditioned fear in rats. Behavioral neuroscience 109, 681 (1995). 7576212 10.1037//0735-7044.109.4.681

[b17] Quirk G. J. Memory for extinction of conditioned fear is long-lasting and persists following spontaneous recovery. Learning & Memory 9, 402–407 (2002). 12464700 10.1101/lm.49602 PMC187587

[b18] Hartley C. A., Fischl B. & Phelps E. A. Brain structure correlates of individual differences in the acquisition and inhibition of conditioned fear. Cerebral Cortex 21, 1954–1962 (2011). 21263037 10.1093/cercor/bhq253 PMC3155599

[b19] Milad M. R. . Thickness of ventromedial prefrontal cortex in humans is correlated with extinction memory. Proceedings of the National Academy of Sciences of the United States of America 102, 10706 (2005). 16024728 10.1073/pnas.0502441102 PMC1180773

[b20] Delgado M. R., Nearing K. I., LeDoux J. E. & Phelps E. A. Neural circuitry underlying the regulation of conditioned fear and its relation to extinction. Neuron 59, 829–838 (2008). 18786365 10.1016/j.neuron.2008.06.029 PMC3061554

[b21] Xue Y. X. . A Memory Retrieval-Extinction Procedure to Prevent Drug Craving and Relapse. Science 336, 241–245 (2012). 22499948 10.1126/science.1215070 PMC3695463

[b22] Quirk G. J. & Milad M. R. Neuroscience: Editing out fear. Nature 463, 36–37 (2010). 20054384 10.1038/463036a

[b23] Akirav I. & Maroun M. Ventromedial prefrontal cortex is obligatory for consolidation and reconsolidation of object recognition memory. Cerebral Cortex 16, 1759–1765 (2006). 16421330 10.1093/cercor/bhj114

[b24] Nader K., Schafe G. E. & Le Doux J. E. Fear memories require protein synthesis in the amygdala for reconsolidation after retrieval. Nature 406, 722–726 (2000). 10963596 10.1038/35021052

[b25] Duvarci S. & Nader K. Characterization of fear memory reconsolidation. The Journal of Neuroscience 24, 9269–9275 (2004). 15496662 10.1523/JNEUROSCI.2971-04.2004 PMC6730081

[b26] Graham B. M. & Richardson R. Intraamygdala infusion of fibroblast growth factor 2 enhances extinction and reduces renewal and reinstatement in adult rats. The Journal of Neuroscience 31, 14151–14157 (2011). 21976500 10.1523/JNEUROSCI.3014-11.2011 PMC6623635

[b27] Bouton M. E. Context, ambiguity, and unlearning: sources of relapse after behavioral extinction. Biological psychiatry 52, 976–986 (2002). 12437938 10.1016/s0006-3223(02)01546-9

[b28] Dudai Y. Reconsolidation: the advantage of being refocused. Current opinion in neurobiology 16, 174–178 (2006). 16563730 10.1016/j.conb.2006.03.010

[b29] Nader K. & Hardt O. A single standard for memory: the case for reconsolidation. Nature Reviews Neuroscience 10, 224–234 (2009). 19229241 10.1038/nrn2590

[b30] Barak S. & Hamida S. B. Memory erasure, enhanced extinction and disrupted reconsolidation. The Journal of Neuroscience 32, 2250–2251 (2012). 22396400 10.1523/JNEUROSCI.6123-11.2012 PMC6621822

[b31] Andrews-Hanna J. R. . Disruption of large-scale brain systems in advanced aging. Neuron 56, 924–935 (2007). 18054866 10.1016/j.neuron.2007.10.038 PMC2709284

[b32] Greicius M. D., Supekar K., Menon V. & Dougherty R. F. Resting-state functional connectivity reflects structural connectivity in the default mode network. Cerebral Cortex 19, 72–78 (2009). 18403396 10.1093/cercor/bhn059 PMC2605172

[b33] Hagmann P. . Mapping the structural core of human cerebral cortex. PLoS biology 6, e159 (2008). 18597554 10.1371/journal.pbio.0060159 PMC2443193

[b34] Vincent J. . Intrinsic functional architecture in the anaesthetized monkey brain. Nature 447, 83–86 (2007). 17476267 10.1038/nature05758

[b35] Biswal B., Yetkin F. Z., Haughton V. M. & Hyde J. S. Functional connectivity in the motor cortex of resting human brain using echo-planar MRI. Magnetic resonance in medicine 34, 537–541 (1995). 8524021 10.1002/mrm.1910340409

[b36] Kiviniemi V. . Slow vasomotor fluctuation in fMRI of anesthetized child brain. Magnetic resonance in medicine 44, 373–378 (2000). 10975887 10.1002/1522-2594(200009)44:3<373::aid-mrm5>3.0.co;2-p

[b37] Zang Y.-F. . Altered baseline brain activity in children with ADHD revealed by resting-state functional MRI. Brain and Development 29, 83–91 (2007). 16919409 10.1016/j.braindev.2006.07.002

[b38] Agren T. . Disruption of Reconsolidation Erases a Fear Memory Trace in the Human Amygdala. Science 337, 1550–1552 (2012). 22997340 10.1126/science.1223006

[b39] Etkin A., Egner T. & Kalisch R. Emotional processing in anterior cingulate and medial prefrontal cortex. Trends in cognitive sciences 15, 85–93 (2011). 21167765 10.1016/j.tics.2010.11.004 PMC3035157

[b40] Alberini C. M. Mechanisms of memory stabilization: are consolidation and reconsolidation similar or distinct processes? Trends in neurosciences 28, 51–56 (2005). 15626497 10.1016/j.tins.2004.11.001

[b41] Hupbach A., Gomez R., Hardt O. & Nadel L. Reconsolidation of episodic memories: a subtle reminder triggers integration of new information. Learning & Memory 14, 47–53 (2007). 17202429 10.1101/lm.365707 PMC1838545

[b42] Frysztak R. J. & Neafsey E. J. The effect of medial frontal cortex lesions on respiration,“freezing,” and ultrasonic vocalizations during conditioned emotional responses in rats. Cerebral Cortex 1, 418–425 (1991). 1822749 10.1093/cercor/1.5.418

[b43] Corcoran K. A. & Quirk G. J. Activity in prelimbic cortex is necessary for the expression of learned, but not innate, fears. The Journal of neuroscience 27, 840–844 (2007). 17251424 10.1523/JNEUROSCI.5327-06.2007 PMC6672908

[b44] LaBar K. S., Gatenby J. C., Gore J. C., LeDoux J. E. & Phelps E. A. Human amygdala activation during conditioned fear acquisition and extinction: a mixed-trial fMRI study. Neuron 20, 937–945 (1998). 9620698 10.1016/s0896-6273(00)80475-4

[b45] Kalisch R., Wiech K., Critchley H. D. & Dolan R. J. Levels of appraisal: a medial prefrontal role in high-level appraisal of emotional material. Neuroimage 30, 1458–1466 (2006). 16388969 10.1016/j.neuroimage.2005.11.011

[b46] Bechara A., Damasio H. & Damasio A. R. Emotion, decision making and the orbitofrontal cortex. Cerebral Cortex 10, 295–307 (2000). 10731224 10.1093/cercor/10.3.295

[b47] Schiller D. & Delgado M. R. Overlapping neural systems mediating extinction, reversal and regulation of fear. Trends in cognitive sciences 14, 268–276 (2010). 20493762 10.1016/j.tics.2010.04.002 PMC3848321

[b48] Mauss I. B., Bunge S. A. & Gross J. J. Automatic emotion regulation. Social and Personality Psychology Compass 1, 146–167 (2007).

[b49] Carretié L., Hinojosa J. A., Mercado F. & Tapia M. Cortical response to subjectively unconscious danger. Neuroimage 24, 615–623 (2005). 15652297 10.1016/j.neuroimage.2004.09.009

[b50] Westen D., Blagov P. S., Harenski K., Kilts C. & Hamann S. Neural bases of motivated reasoning: An fMRI study of emotional constraints on partisan political judgment in the 2004 US presidential election. Journal of Cognitive Neuroscience 18, 1947–1958 (2006). 17069484 10.1162/jocn.2006.18.11.1947

[b51] Margulies D. S. . Mapping the functional connectivity of anterior cingulate cortex. Neuroimage 37, 579–588 (2007). 17604651 10.1016/j.neuroimage.2007.05.019

[b52] Tronson N. C., Wiseman S. L., Olausson P. & Taylor J. R. Bidirectional behavioral plasticity of memory reconsolidation depends on amygdalar protein kinase A. Nature neuroscience 9, 167–169 (2006). 16415868 10.1038/nn1628

[b53] Schiller D., Kanen J. W., LeDoux J. E., Monfils M.-H. & Phelps E. A. Extinction during reconsolidation of threat memory diminishes prefrontal cortex involvement. Proceedings of the National Academy of Sciences 110, 20040–20045 (2013). 10.1073/pnas.1320322110 PMC3864277 24277809

[b54] Simpson J. R., Drevets W. C., Snyder A. Z., Gusnard D. A. & Raichle M. E. Emotion-induced changes in human medial prefrontal cortex: II. During anticipatory anxiety. Proceedings of the National Academy of Sciences 98, 688–693 (2001). 10.1073/pnas.98.2.688 PMC14649 11209066

[b55] Dębiec J., Bush D. E. & LeDoux J. E. Noradrenergic enhancement of reconsolidation in the amygdala impairs extinction of conditioned fear in rats—a possible mechanism for the persistence of traumatic memories in PTSD. Depression and anxiety 28, 186–193 (2011). 21394851 10.1002/da.20803 PMC3590026

[b56] Raichle M. E. The brain's dark energy. SCIENCE-NEW YORK THEN WASHINGTON - 314, 1249 (2006). 17124311

[b57] Motzkin J. C., Philippi C. L., Wolf R. C., Baskaya M. K. & Koenigs M. Ventromedial prefrontal cortex is critical for the regulation of amygdala activity in humans. Biological psychiatry 77, 276–284 (2015). 24673881 10.1016/j.biopsych.2014.02.014 PMC4145052

[b58] Gold A. L., Morey R. A. & McCarthy G. Amygdala–prefrontal cortex functional connectivity during threat-induced anxiety and goal distraction. Biological psychiatry 77, 394–403 (2015). 24882566 10.1016/j.biopsych.2014.03.030 PMC4349396

[b59] Lang P. J., Bradley M. M. & Cuthbert B. N. (Gainesville, FL: The Center for Research in Psychophysiology, University of Florida, 1999).

[b60] Friston K. J. . Statistical parametric maps in functional imaging: a general linear approach. Human brain mapping 2, 189–210 (1994).

[b61] Yan & Zang. DPARSF: a MATLAB toolbox for “pipeline” data analysis of resting-state fMRI. Frontiers in systems neuroscience 4, 13 (2010). 20577591 10.3389/fnsys.2010.00013 PMC2889691

[b62] Song X. W. . REST: a toolkit for resting-state functional magnetic resonance imaging data processing. PloS one 6, e25031 (2011). 21949842 10.1371/journal.pone.0025031 PMC3176805

[b63] Lowe M., Mock B. & Sorenson J. Functional connectivity in single and multislice echoplanar imaging using resting-state fluctuations. Neuroimage 7, 119–132 (1998). 9558644 10.1006/nimg.1997.0315

[b64] Brett M., Anton J.-L., Valabregue R. & Poline J.-B. Region of interest analysis using the MarsBar toolbox for SPM 99. Neuroimage 16, S497 (2002).

[b65] Fox M. D. . The human brain is intrinsically organized into dynamic, anticorrelated functional networks. Proceedings of the National Academy of Sciences of the United States of America 102, 9673 (2005). 15976020 10.1073/pnas.0504136102 PMC1157105

[b66] Roy A. K. . Functional connectivity of the human amygdala using resting state fMRI. Neuroimage 45, 614–626 (2009). 19110061 10.1016/j.neuroimage.2008.11.030 PMC2735022

[b67] Di Martino A. . Functional connectivity of human striatum: a resting state FMRI study. Cerebral cortex 18, 2735–2747 (2008). 18400794 10.1093/cercor/bhn041

